# Cleidocranial Dysplasia: A Case Report

**DOI:** 10.4274/jcrpe.v2i3.134

**Published:** 2010-08-09

**Authors:** Gülay Karagüzel, Filiz Azar Aktürk, Emelgül Okur, Halit Reşit Gümele, Yusuf Gedik, Ayşenur Ökten

**Affiliations:** 1 Karadeniz Technical University, School of Medicine, Department of Pediatric Endocrinology, Trabzon, Türkiye; 2 Karadeniz Technical University, School of Medicine Department of Pediatrics, Trabzon, Türkiye; 3 Karadeniz Technical University, School of Medicine Department of Radiology, Trabzon, Türkiye; +90 462 377 59 24+90 462 377 54 73gulaykg@yahoo.comKaradeniz Technical University, School of Medicine, Department of Pediatric Endocrinology, Trabzon, Türkiye

**Keywords:** Cleidocranial dysplasia, unclosed fontanelle, aplasia of clavicle

## Abstract

Cleidocranial dysplasia (CCD) is a rare autosomal dominant skeletal disease. CCD is caused by mutation in the gene on 6p21 encoding transcription factor CBFA1, i.e. runt−related transcription factor 2(RUNX2). The disease is characterized by a persistently open anterior fontanelle and skull sutures, hypoplastic or aplastic clavicles, dental abnormalities, short stature, a wide pubic symphysis, and a variety of other skeletal changes. A major finding of CCD is hypoplasia or aplasia of clavicular bones resulting in the ability of the patient to approximate the shoulders. Delayed closure of the anterior fontanelle and of metopic sutures causes frontal bossing. We report a case of CCD in a 3.5−yearold boy who referred to our clinic because of an unclosed anterior fontanelle and emphasize the importance of clinical findings in CCD.

**Conflict of interest:**None declared.

## INTRODUCTION

Cleidocranial dysplasia (CCD) is a rare dominantly inherited autosomal bone disease that is characterized by delayed closure of fontanelles, presence of open skull sutures, hypoplastic or aplastic clavicles, supernumerary teeth, delayed eruption of permanent dentition, wide pubic symphysis, short stature and a variety of other skeletal changes. Delayed closure of the anterior fontanelle and metopic sutures results in frontal bossing. The phenotypic spectrum ranges from mildly affected individuals with dental anomalities only to severely affected patients with syringomyelia (1, 2). CCD is also known as Marie−Sainton disease, mutational dysostosis, and cleidocranial dysostosis (3). Human osteoblast−specific, runt−related transcription factor 2 (RUNX2) gene located on chromosome 6p21 is identified as the gene responsible for CCD (4).

Here, we report a case of CCD in a 3.5−year−old boy and emphasize the importance of clinical examination findings.

## CASE REPORT

A 3.5−year−old boy was referred to our clinic because of unclosed anterior fontanelle. He was born at 40 weeks gestation to healthy parents, and his birth weight was 3300 g. He started walking at age 11 months.

**Figure 1:**
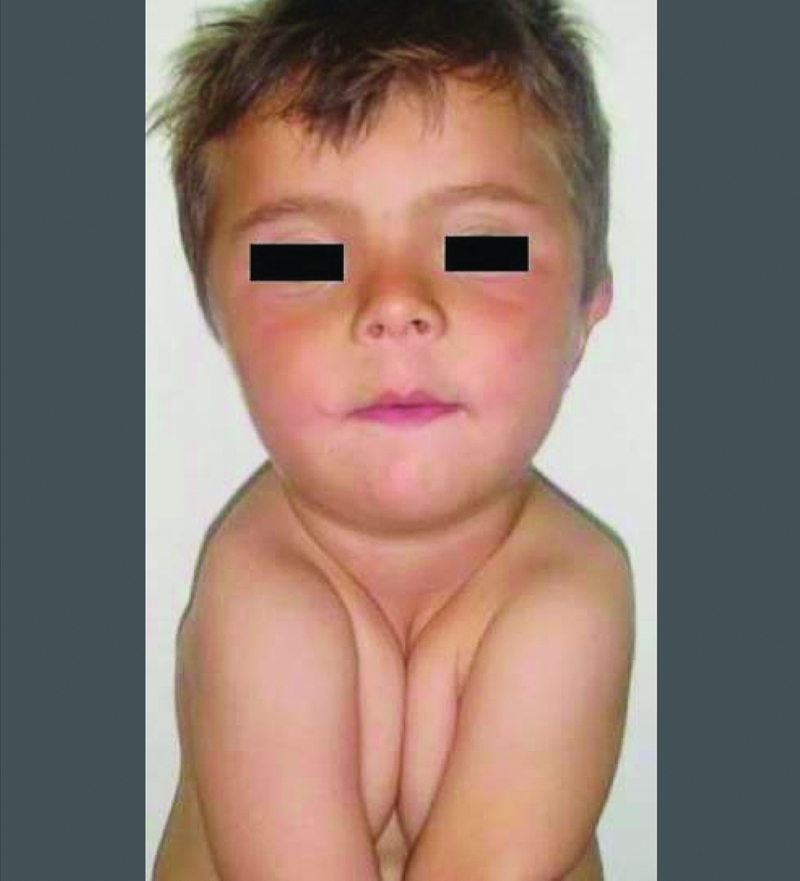
Facial appreance and hypermobile shoulders

Physical examination revealed a weight of 15 kg (25−50^th^ percentile), height of 92 cm (3−10^th^ percentile), and head circumference of 53 cm (50^th^ percentile). The anterior fontanelle was open, with vertical and horizontal diameters of 4 cm and 3 cm, respectively. A high−arched palate, low nasal bridge, dental deformities, hyperodontia, mandibular retrognathism, brachiocephalic head and face were also noted. The right clavicle was absent, the shoulders were ptotic and hypermobile (Figure 1). Other system examinations were normal. The family history revealed no other member with bony abnormalities, delayed ossification, or short stature.

Laboratory investigations showed normal serum calcium, phosphorus, alkaline phosphatase, parathyroid hormone and vitamin D levels. Thyroid function tests were within normal ranges.

**Figures 2a:**
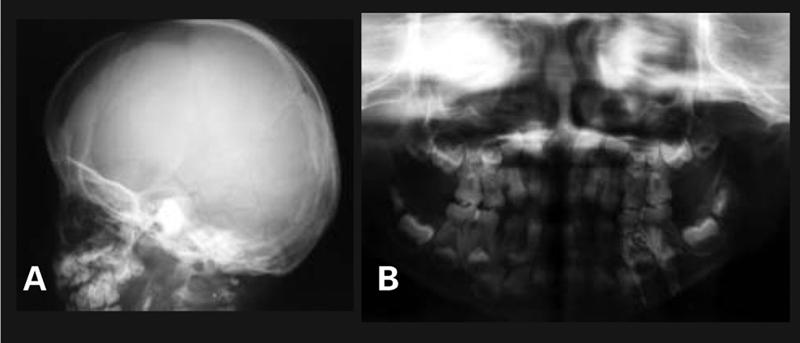
Large anterior fontanelle, wormian bones, sclerotic skull base

**Figures 3a:**
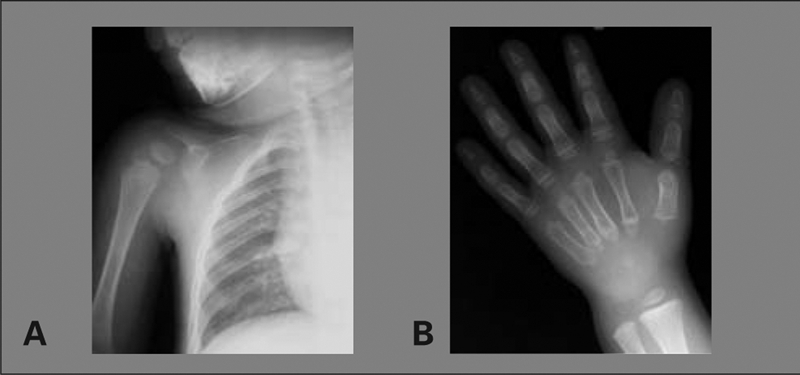
Aplasia of the right clavicle

**Figure 4:**
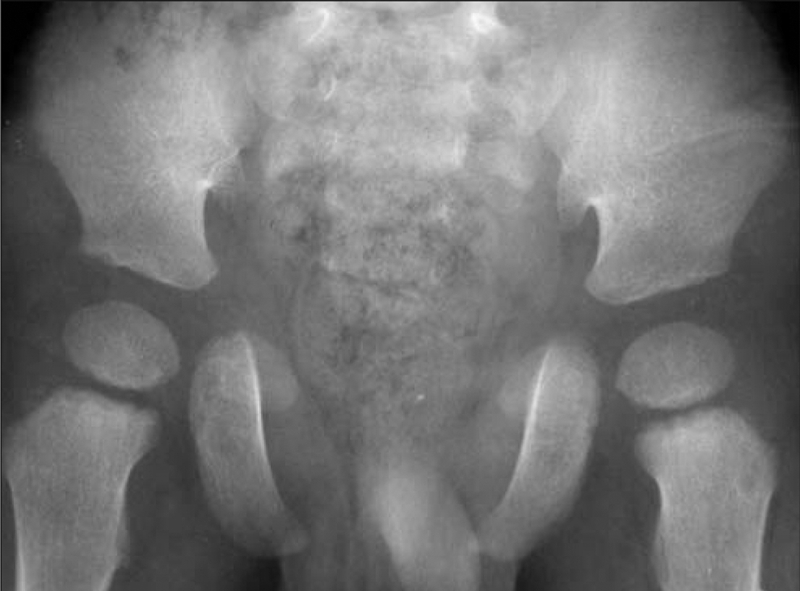
Wide pubic symphysis and coxa vara

Bone age was 4 years. Bone radiography demonstrated a large anterior fontanelle, wormian bones, a sclerotic skull base, multiple supernumerary teeth and malocclusion (Figures 2a and 2b), aplasia of the right clavicle and a hypoplastic left clavicle, a narrow chest, hypoplastic distal phalanges, cone−shaped epiphyses of middle phalanges (Figures 3a and 3b), a wide pubic symphysis, and coxa vara (Figure 4).

Based on these clinical and radiological findings, the patient was diagnosed as a case of CCD.

**2b:**
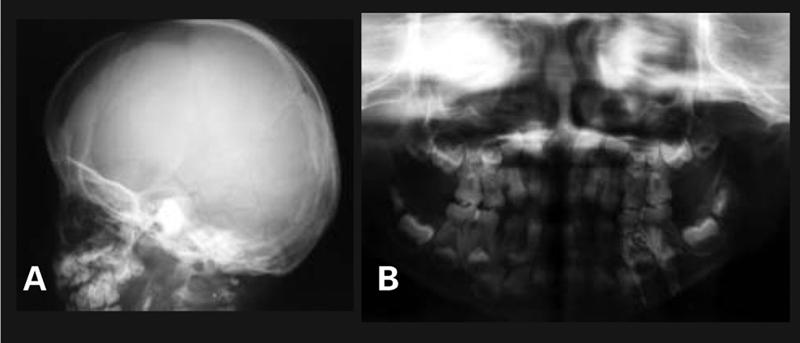
Multiple supernumerary teeth

**3b:**
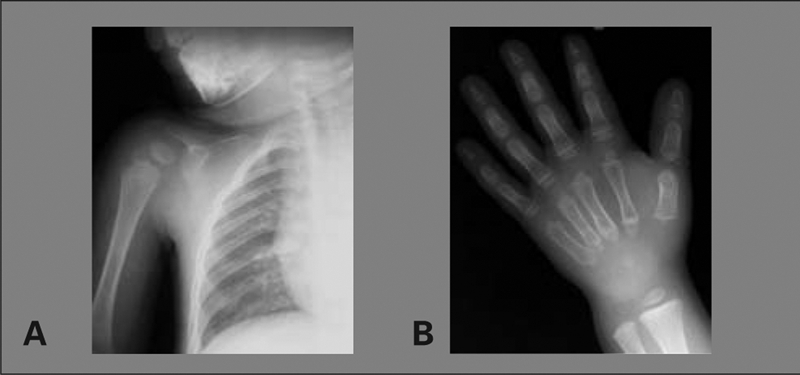
Hypoplasia of distal phalanges and cone−shaped epiphyses of middle phalanges

## DISCUSSION

The major features of CCD are aplastic or hypoplastic clavicles, dental abnormalities (multiple supernumerary teeth, multiple impacted permanent teeth, retention of the deciduous teeth), and delayed closure of the sagittal fontanelles. Typically, our patient had all of these findings that are pathognomonic for a diagnosis of CCD (1). Other findings of CCD are short stature, a bell−shaped thorax, hypoplasia of the pelvis, enlargement of the frontal and occipital bones, and phalangeal abnormalities. Shortened or absent nasal bones, paranasal sinus abnormalities, thickening of some segments of the calvaria, small maxillae, and delayed union of the mandibular symphysis are less common findings of CCD. There is a notably phenotypic variation of CCD even within one and the same family. In approximately 40% of CCD patients, a genetic transition cannot be identified, and the condition develops spontaneously (1, 5, 6, 7).

Clavicules are underdeveloped to varying degrees in these patients and are completely absent in approximately 10 percent. This allows excessive mobility of the shoulder girdle, as was also observed in our patient.

Dental abnormality is one of the main features of CCD. Our patient had multiple supernumerary teeth, which can impede the normal eruption of permanent teeth. It has been suggested that supernumerary teeth in such cases should be removed as soon as possible (6, 7, 8).

The main finding in our patient was an open anterior fontanelle. Delayed closure of fontanelles could be a feature of hypothyroidism, rickets, hypophosphatasia, osteogenesis

imperfecta, pycnodysostosis, and other syndromes such as Apert syndrome, Dubowitz syndrome, Russell−Silver syndrome, Down’s syndrome, and Crouzon syndrome (10). When other characteristic features are taken into account, CCD can be differentiated easily from the other skeletal disorders and syndromes.

It is known that CCD is caused by heterozygous mutations in RUNX2 gene, which encodes a transcription factor required for osteoblast differentiation and is located on chromosome 6p21 (1, 9). Many mutations in the RUNX2 gene have been identified in patients with CCD.

In conclusion, CCD should be kept in mind by pediatricians as a cause of delayed closure of the anterior fontanelle. Although the clinical findings of CCD are present at birth, diagnosis of the disease is often delayed. Thus, we want once again to draw attention to the importance of physical examination in the diagnosis of this disease.
